# *Bacillus licheniformis* Alleviates DSS-Induced Ulcerative Colitis in Mice by Repairing the Intestinal Barrier and Regulating Gut Microbiota and Its Metabolism

**DOI:** 10.3390/nu18081311

**Published:** 2026-04-21

**Authors:** Hongwei Ma, Mengen Xu, Ying Yu, Ziyi Xia, Muhammad Farhan Rahim, Min A, Ziyang Wang, Chengxu Xu, Jiakui Li

**Affiliations:** 1 College of Veterinary Medicine, Huazhong Agricultural University, Wuhan 430070, China; mahongwei100@126.com (H.M.); xme183@mail.hzau.edu.cn (M.X.); 13984993325@163.com (Y.Y.); xiazy2019@webmail.hzau.edu.cn (Z.X.); farhan092@webmail.hzau.edu.cn (M.F.R.); cocominlora@outlook.com (M.A.); hzauy514@163.com (Z.W.); gologic@163.com (C.X.); 2 Hubei Jiangxia Laboratory, Wuhan 430200, China; 3 College of Animals Husbandry and Veterinary Medicine, Tibet Agricultural and Animal Husbandry University, Nyingchi 860000, China

**Keywords:** ulcerative colitis, *Bacillus licheniformis*, intestinal barrier, oxidative stress, gut microbiota homeostasis, microbial metabolism

## Abstract

Background: Inflammatory bowel disease (IBD) is a gut-based idiopathic disease characterized by chronic and relapsing inflammatory progression and intricate pathophysiology. It is now known that the key etiologies of IBD include immune dysregulation, imbalances in the gut microbiota, and metabolic disruptions. Probiotics are now the potential treatment for IBD, due to their ability to regulate the host immune system and microbiota of the gut. Methods: The current study analytically tested the preventive benefit of *Bacillus licheniformis* BL-01 on dextran sulfate sodium (DSS)-induced ulcerative colitis (UC) and also expounded on its molecular pathogenesis. Results: Our results demonstrate that supplementation with BL-01 effectively mitigates DSS-induced weight loss, an elevated disease activity index (DAI), and colonic tissue injury in mice. Concomitantly, BL-01 rectifies dysregulated inflammatory cytokine profiles, attenuates oxidative stress, and restores the expression of colonic tight junction proteins as well as the number of goblet cells. Furthermore, BL-01 modulates the gut microbiota diversity by increasing the abundance of beneficial bacterial genera such as *Duncaniella* and decreasing the abundance of pathogenic genera such as *Helicobacter*. Notably, BL-01 restores DSS-induced microbial metabolic dysregulation, modulates key metabolic pathways including arachidonic acid metabolism and steroid hormone biosynthesis, and regulates associated metabolites to ameliorate UC. Finally, *Bacillus licheniformis* BL-01 mitigates oxidative stress, reverses gut dysbiosis and metabolic disorders, and has a protective effect on UC. Conclusions: The findings give new information on the development of probiotic-based therapeutics in the prevention and treatment of IBD.

## 1. Introduction

Inflammatory bowel disease (IBD) is chronic, relapsing inflammation of the intestine, which is mainly manifested in the form of ulcerative colitis (UC) and Crohn’s disease (CD) and is caused by the sustained nonspecific inflammation of the gastrointestinal tract [1]. Unlike CD, which may attack the whole thickness of the gastrointestinal tract and the whole digestive system, the lesions of UC are usually limited to the mucosal and submucosal layers of the colon and rectum. It is characterized by clinical manifestations, including chronic or recurrent abdominal pain, diarrhea, and hematochezia [2]. Currently, the global incidence of IBD is on the rise, which is a trend that is particularly prominent in developing countries that imposes a substantial social burden [3]. Although the pathogenesis of IBD remains incompletely elucidated, the combined effects of genetic predisposition, gut microbiota dysbiosis, immune dysfunction, and environmental factors are widely recognized as being the primary etiological factors [4]. Aminosalicylates, glucocorticoids, and immunosuppressants are currently the standard clinical therapies for mild to moderate IBD. However, due to the unclear pathogenesis, these therapeutic approaches exhibit limited efficacy and are associated with certain adverse effects [5]. Consequently, researchers are striving to explore more effective and safer therapeutic strategies, including stem cell therapy, fecal microbiota transplantation, plant extract therapy, and novel microbial preparations [6]. Notably, there is a growing interest in microbial preparations, which is primarily due to their natural origin, established safety, and demonstrated efficacy.

The gut microbiota is sometimes called a hidden organ, based on a symbiotic relationship that exists between the microbiota and its hosts via complex networks and cross-talk interactions. It maintains intestine health by maintaining the intestinal mucosal barrier, stabilizing the immune system, and balancing intestinal metabolism [7]. Accumulating evidence has demonstrated that the gut microbiota of patients diagnosed with IBD is characterized by reduced diversity and altered composition. Consequently, the modulation of gut microbiota dysbiosis may emerge as a novel therapeutic strategy for IBD [8]. Probiotics, defined as live microorganisms that can colonize the host’s gut when administered in adequate amounts to regulate the intestinal microbiota, have spurred extensive research into their potential as a therapeutic approach for IBD [9]. For instance, Liu et al. demonstrated that *Lactobacillus acidophilus* C4 can alleviate DSS-induced colitis in mice by repairing the intestinal mucosal barrier and restoring gut microbiota diversity [10]. Zheng et al. also demonstrated that *Bifidobacterium acidifaciens* and its derived extracellular vesicles can alleviate colitis by reducing inflammatory responses, restoring gut microbiota diversity, and rebalancing the gut microbial composition [11]. However, owing to inherent strain-specific variations and the effects of individual genetics, dietary patterns, and baseline gut microbiomes, the efficacy of different probiotic strains varies significantly. This represents a critical consideration when selecting probiotics as therapeutic agents for intestinal disorders [12].

*Bacillus* spp., a genus of facultatively anaerobic Gram-positive bacteria, exhibits high thermal stability, a trait attributed to its spore-forming ability. This genus plays a vital role in maintaining gut microbiota homeostasis, promoting nutrient absorption, and inhibiting potential pathogens [13]. Previous studies have confirmed that *Bacillus* spp. can increase the length of intestinal villi in the mouse mucosal epithelium, thereby enlarging the intestinal epithelial surface area and enhancing nutrient absorption [14]. These beneficial probiotic effects are closely associated with their ability to regulate the expression of vitamins, amino acids, and growth-promoting factors in the gut, thereby exerting favorable impacts on the host. Accordingly, *Bacillus* has also been used as a safe probiotic supplement in aquaculture. Zeng et al. reported that *Bacillus licheniformis* isolated from yaks improved daily weight gain and feed conversion efficiency in mice [15]. Li et al. also demonstrated that Bacillus subtilis and Bacillus brevis exert excellent growth promoting effects, as well as anti-inflammatory and antioxidant activities in mice [16]. *Bacillus* spp. have been shown to confer antibacterial effects by consuming intestinal oxygen, thereby inhibiting the proliferation of harmful aerobic bacteria [17]. Meanwhile, the antimicrobial peptides and short-chain fatty acids produced by *Bacillus* spp. can suppress the growth of pathogenic bacteria, stimulate intestinal SIgA secretion, promote immune organ development, and enhance host immunity [18]. Dong et al. verified that *Bacillus subtilis* BYS2 enhances the antibacterial and antiviral capacity of broilers by elevating the serum IgG and IgM levels [19]. Collectively, the multiple biological properties of *Bacillus* spp. suggest their considerable therapeutic potential for intestinal diseases. However, most previous studies have focused on their protective effects against infectious intestinal diseases, while their effects on IBD remain poorly understood [20]. In the present study, we investigated the preventive efficacy and underlying molecular mechanisms of *Bacillus licheniformis* in DSS-induced colitis, aiming to further expand the application scope of *Bacillus* spp. and provide new insights for the development of therapeutic strategies against IBD.

## 2. Materials and Methods

### 2.1. Animals and Experimental Strain

The present study was approved by the Institutional Animal Care and Ethics Committee of Huazhong Agricultural University. The Experimental Animal Center of Huazhong Agricultural University acquired 36 male C57BL/6 mice aged 6 weeks and allowed them to freely access food and water. The strain of *Bacillus licheniformis* BL-01 that was used in this study was obtained from the rumen content of Zaobei cattle in the Province of Hubei, China, which was kept in our laboratory. The taxonomic status of BL-01 was identified by whole-genome sequencing. The sequencing data generated in this study have been submitted to the NCBI database under BioProject accession number PRJNA1449211. Bacterial cells were incubated in LB medium (Solarbio, Beijing, China) for 12 h at 37 °C and then centrifuged at 8000 rpm for 10 min to obtain the bacterial pellet. After rigorous washing using sterile PBS (Biosharp, Beijing, China), the concentration of the cells of the bacterium BL-01 was set to 1.0 × 10^9^ CFU/mL to conduct the subsequent experiment.

### 2.2. Animal Experiments

Following acclimatization for one week, mice were randomly assigned to three groups (*n* = 12 per group) using a random number table: Control (CT), DSS, and BL-DSS [12,21]. During the initial phase of the experiment (days 0–14), the BL-DSS group was given a daily oral gavage of 1 × 10^9^ CFU BL-01, whereas the CT and DSS groups received an equal volume of PBS. From day 14 to day 21, 2.5% DSS solution (MP Biomedicals, Santa Ana, CA, USA) was provided in the drinking water for the DSS and BL-DSS groups to induce UC [12,21]. The body weight, health status (including diarrhea, bloody stools, and general demeanor), and mortality of the mice were closely monitored and recorded daily. The Disease Activity Index (DAI) was used to assess the severity of colitis, with scores being calculated based on weight loss, diarrhea, and hematochezia [22,23]. On day 21 of the experiment, all mice were deeply anesthetized with isoflurane prior to euthanasia by cervical dislocation. The serum was obtained by centrifuging the collected blood samples and then preserved at −80 °C until use. After recording the colon length, the colon tissue was dissected into two parts. The distal portion was immersed in 4% paraformaldehyde for fixation, and the remainder was immediately frozen at −80 °C. All housing, treatments, and sample collection were standardized to minimize confounding factors. Investigators involved in outcome assessment and data analysis were blinded to group allocation.

### 2.3. Histological Analysis and Immunohistochemistry

For the purpose of a histological assessment of the mouse colon tissue, it is first necessary to fix the specimens in 4% paraformaldehyde for a period of 48 h. Following this, the specimens undergo a process of ethanol gradient dehydration and paraffin embedding. Sections of 5 μm thickness were prepared and subsequently stained with hematoxylin and eosin. In addition, we employed AB-PAS staining to analyze the number of goblet cells present in the fixed colonic tissue. In order to undertake a comprehensive evaluation of the expression levels of tight junction proteins (ZO-1 and Occludin), a range of immunohistochemical staining techniques were employed on the colonic tissue samples. Finally, the images were captured using an optical microscope.

### 2.4. Measurement of Inflammatory Cytokines and Antioxidant Enzymes in Serum

In order to assess the systemic inflammation and oxidative stress levels in mice, appropriate ELISA kits (Jiancheng, Nanjing, China) were used to measure inflammatory cytokines and antioxidant enzymes in serum. The procedure was performed in strict accordance with the manufacturer’s instructions provided with the kits. The inflammatory cytokines detected primarily include tumor necrosis factor (TNF-α), interferon-γ (IFN-γ), interleukin-1β (IL-1β), interleukin-4 (IL-4), interleukin-6 (IL-6), and interleukin-10 (IL-10). The antioxidant markers detected in this study include malondialdehyde (MDA), glutathione peroxidase (GSH-Px), total superoxide dismutase (T-SOD), and catalase (CAT) [21].

### 2.5. Extraction, Sequencing, and Analysis of DNA from Intestinal Contents

The total genomic DNA of the microbial community of the mouse cecal contents was isolated using a QIAamp DNA Mini Kit (Qiagen, Dusseldorf, Germany). Agarose gel electrophoresis was then used to determine the quality of the extracted DNA, after which its concentration and purity were determined by using a UV spectrophotometer (Thermo Scientific, Waltham, MA, USA). PCR amplification was done by using standard primers to the V3/V4 hypervariable of the bacterial 16S rRNA gene (338F: ACT CCT ACG GGA GGC AGCA; 806R: GGA CTA CHV GGG TWT CTA AT) [24]. The PCR products underwent agarose gel electrophoresis, after which the target gene fragments were purified and rescued with Vazyme VAHTSTM DNA Clean Beads. The recovered products were then quantified by the means of a fluorescent quantification assay on a microplate reader (FLx800, BioTek, Winooski, VT, USA). Upon the result analysis and identification of the sequencing parameters needed, the samples were mixed in the right proportions. Illumina MiSeq was used to perform the high-throughput sequencing of quality-checked samples constructed using the Illumina TruSeq Nano DNA LT Library Prep Kit (Illumina, San Diego, CA, USA). The acquired sequences were clustered into operational taxonomic units (OTUs) with UPARSE (version 7.1), applying a 97% sequence similarity cutoff. Alpha diversity indices were subsequently calculated from the resulting OTU profiles, using Mothur (version 1.30.1). To evaluate the similarity of microbial community structures across different samples, principal coordinate analysis (PCoA) was performed with the Vegan package (version 2.5-3). In addition, linear discriminant analysis (LDA), combined with effect size measurement (LEfSe), was utilized to detect differentially abundant taxa ranging from the phylum to genus level among various experimental groups.

### 2.6. Untargeted Metabolomics Analysis of Colonic Contents

The metabolomics analysis was conducted following the procedure reported earlier by Wang et al., comprising sample processing, metabolite detection, and data preprocessing and metabolic pathway identification [25]. The cecal contents (approximately 5 mg) were now blended with pre-chilled methanol, and a zirconia beads homogenate was used at 3 min and incubated at 0 °C 10 min, followed by incubation at −20 °C for 1 h. The centrifugation ended by isolating the supernatant, which was then analyzed by a liquid chromatography–mass spectrometry (LC-MS) system (Waters Corp., Milford, MA, USA). Progenesis QI software was used to extract peptide peaks, align peptide peaks, calibrate, and perform other data processing functions on raw data. It was then determined that metabolites were identified and matched against the METLIN database with Progenesis QI software. Finally, partial least squares discriminant analysis (PLS-DA) and principal component analysis (PCA) were used to visualize the differences in metabolic profiles among groups. Differential metabolites were screened using the criteria of *p* < 0.05 and a variable importance plot (VIP) > 1.

### 2.7. Statistical Analysis

Twelve mice per group were initially allocated to accommodate potential attrition during housing, model induction, treatment, and sample collection. Mice were excluded from the final analysis, based on predefined quality control criteria: outlier values in body weight/DAI, hemolyzed serum samples, and insufficient sample quality for omics detection. Mean ± standard deviation (SD) were used to present all experimental data. The statistical analysis of the comparison across various groups was performed using one-way analysis of variance (ANOVA), as presented by the GraphPad Prism 8.0, where a *p*-value below 0.05 was deemed statistically significant.

## 3. Result

### 3.1. Effects of BL-01 on Physiological Parameters in DSS-Induced Colitis Mice

**Figure 1:**
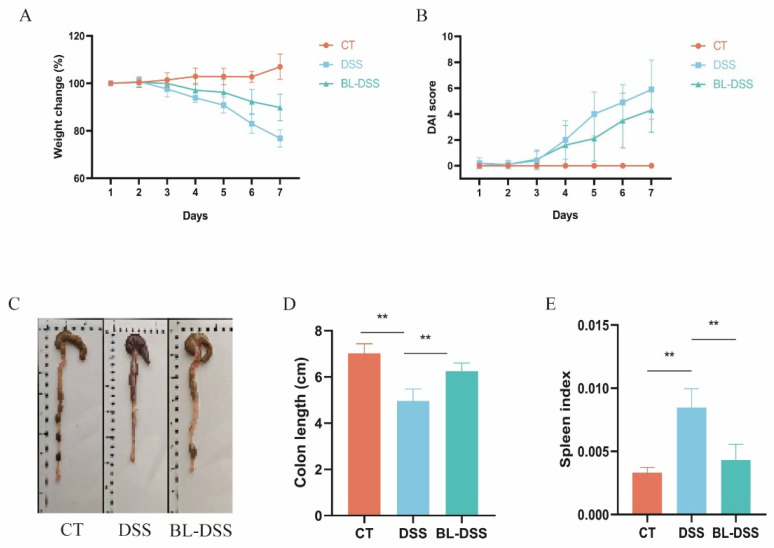
Effects of BL-01 on the parameters of colitis caused by DSS. (**A**) Percent variations in body weight, relative to the initial weight, throughout the period of the experiment. (**B**) The scores of the disease activity index. (**C**,**D**) The length of the colons of mice of each group of the experiment. (**E**) Mice spleen index in each set of experiments. The data are given in the form of means SD. The data is provided in means + SD (*n* = 10), ** *p* < 0.01.

Prior to DSS administration, BL-01 was administered to the mice for 14 days. Administration of BL-01 to the mice failed to cause any toxicity, as gauged by the overall appearance and body weight alteration of the mice. During the next seven days, the drinking water of the DSS and BL-DSS groups received 2.5% DSS to develop colitis, which was indicated by the change in body weight and DAI (Figure 1A,B). Mice in the DSS group exhibited approximately 23% body weight loss, whereas prophylactic supplementation with BL-01 significantly attenuated this weight reduction to only around 10% in the BL-DSS group (Figure 1A). Prophylactic BL-01 treatment also significantly lowered the DAI score compared with the DSS group (Figure 1B). DSS-induced colitis consists of the shortening of the colon. BL-01 supplementation was prophylactic and was effective in reducing the shortening of the colon caused by DSS (Figure 1C,D). It is worth noting that, with the exposure to DSS, the mouse spleen index increased, signifying systemic inflammatory activation. In comparison, this effect was greatly reduced by BL-01 intervention (Figure 1E).

### 3.2. Effects of BL-01 on Colon Histology

**Figure 2:**
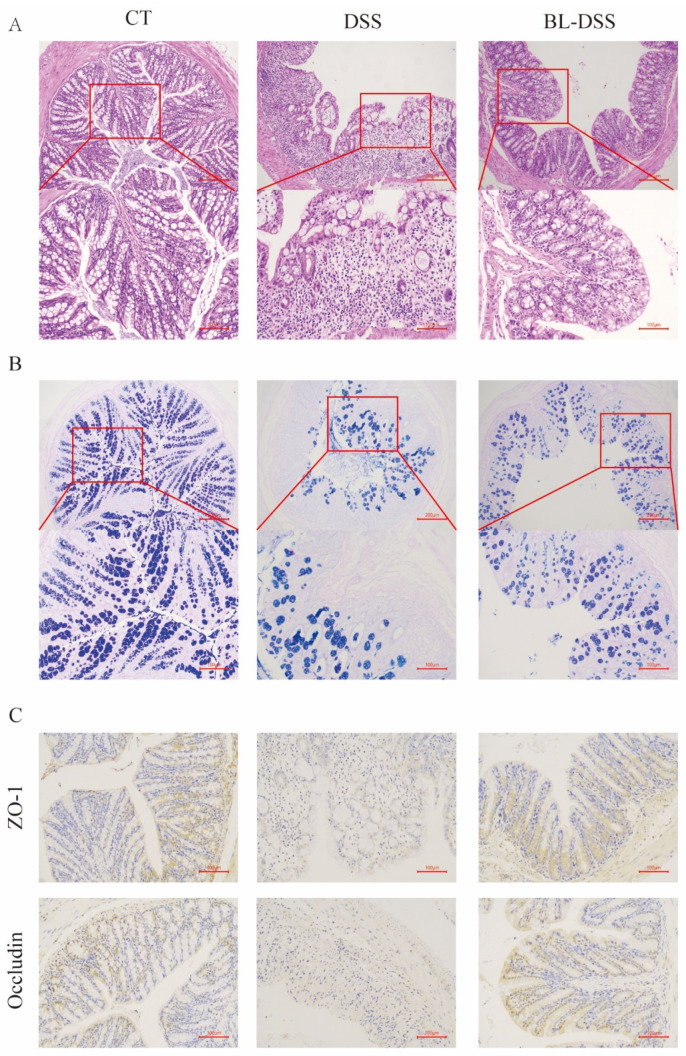
Colon histology effect of BL-01. (**A**) H&E-stained colon sections (100× and 200×). (**B**) Mucin content assessment by AB-PAS (100× and 200×). (**C**) Immunohistochemistry of TJ proteins (200×).

Histological and pathological analysis was done to further determine the protective effect of BL-01 in DSS-induced colonic tissue injury. The effect of BL-01 intervention on the colon morphology was assessed by the use of HE staining. We found that DSS caused considerable inflammatory events in the colon, such as mucosal edema, disruption of the crypt architecture, enormous inflammatory cell invasion, and degeneration of epithelial cells. These pathological alterations were also greatly alleviated in the BL-DSS group (Figure 2A). Colonic goblet cells have a significant role in intestinal defense and therefore, the stain was applied through AB-PAS. The DSS treatment showed a dramatic decrease in the number of goblet cells in the mouse colon compared to BL-01 supplementation that showed a significant restoration of goblet cells (Figure 2B). ZO-1 and Occludin form tight junctions (TJ), which are critical constituents of the intestinal epithelial mechanical barrier. TJ-related proteins were identified by immunohistochemical staining using colonic tissue. DSS treatment reduced the protein content of ZO-1 and Occludin in colonic tissue significantly compared to the CT group (Figure 2C). This effect was significantly reversed in the BL-01 administered mice (Figure 2C). Taken together, these findings indicate that prophylaxis BL-01 supplementation has the ability to protect the colonic mucosal barrier from damage caused by DSS by reducing DSS-induced goblet cell and TJ protein depletion.

### 3.3. Effects of BL-01 on Inflammation and Oxidative Stress Levels in Mice

**Figure 3:**
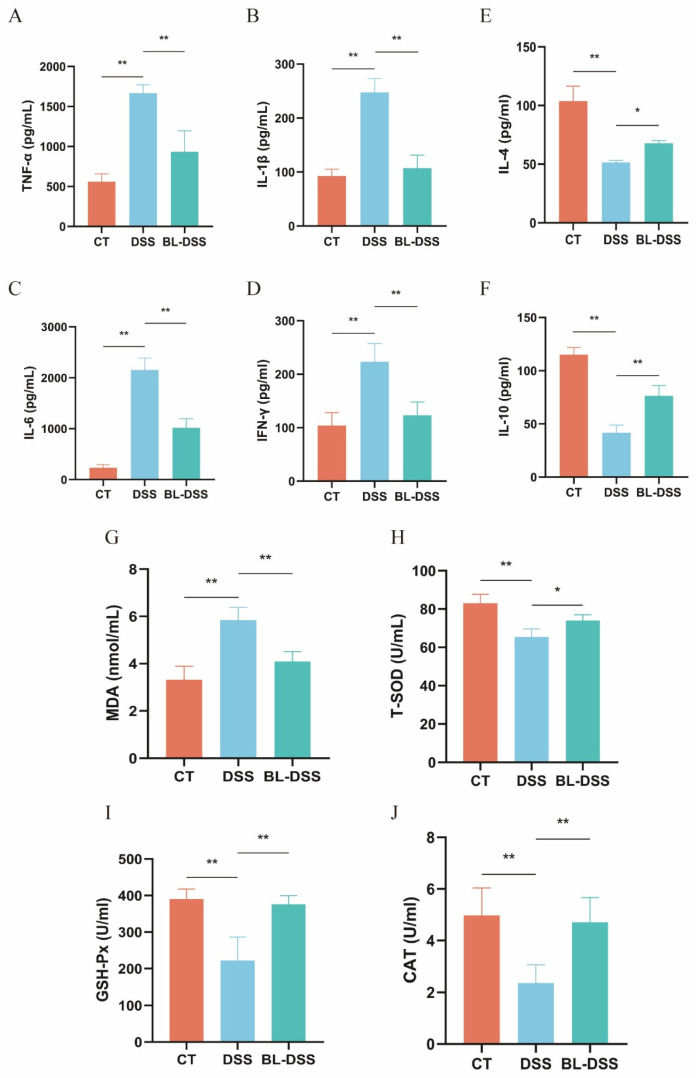
Effect of BL-01 on serum levels of inflammatory factors and oxidative stress in mice. (**A**) TNF-α, (**B**) IL-1β, (**C**) IL-6, (**D**) IFN-γ, (**E**) IL-4, (**F**) IL-10, (**G**) MDA, (**H**) T-SOD, (**I**) GSH-Px, and (**J**) CAT. The data is provided in means + SD (*n* = 8), ** *p* < 0.01, and * *p* < 0.05.

In order to examine the hypothesis that prophylaxis BL-01 supplementation inhibits DSS-induced inflammation and oxidative stress, we analyzed a set of pertinent biomarkers in mice. We found out that DSS therapy increased the serum levels of proinflammatory cytokines (TNF-a, IL-1β, IL-6, and IFN-γ) significantly and decreased the levels of the anti-inflammatory ones (IL-4 and IL-10) significantly. This observation validates the inflammatory reaction of the DSS in murine models. In mice that were administered prophylactic BL-01 supplements prior to the administration of DSS, the pro-inflammatory cytokines were substantially reduced in comparison to the DSS group (Figure 3A–D), whereas the levels of anti-inflammatory cytokines were significantly increased (Figure 3E,F). This agrees with the finding that the prophylactic intake of BL-01 protects goblet cell and tight junction functions, thereby suppressing the penetration of the intestinal microbiota through the mucosal barrier and consequently suppressing the production of proinflammatory cytokines and enhancing the production of anti-inflammatory cytokines, which restores the inflammatory homeostasis. At the same time, serum oxidative stress markers were determined to identify the oxidative stress status. The findings indicated that DSS treatment drastically increased the serum MDA levels, and also drastically decreased the levels of T-SOD, GSH-Px, and CAT. All these abnormalities caused by DSS were greatly reduced in the BL-DSS group (Figure 3G–J). Taken together, the findings demonstrate that BL-01 has a protective effect against colonic inflammation by promoting the activity of antioxidant enzymes and reducing oxidative stress.

### 3.4. Effects of BL-01 on the Composition of the Mouse Gut Microbiota

**Figure 4:**
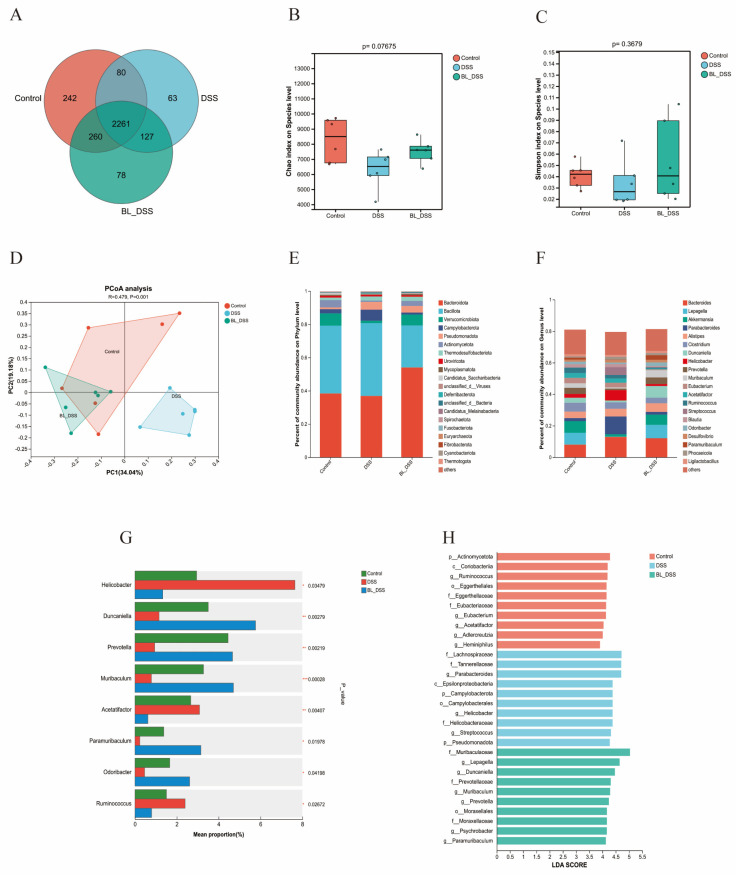
Effects of BL-01 on the intestinal flora of mouse with DSS-induced colitis. (**A**) Venn based on samples’ OTUs. (**B**,**C**) Chao1 index and Simpson index for α-diversity assessment. (**D**) PCoA based on β-diversity metrics. (**E**,**F**) Profiles of the microbial community in the phylum and genus levels. (**G**) Differential abundance analysis showing indicators of greatly changed genera. (**H**) Taxa enriched LDA scores (LDA score threshold = 2). The data is provided in means + SD (*n* = 6). *** *p* < 0.001, ** *p* < 0.01 and * *p* < 0.05.

16S rRNA gene sequencing was performed to analyze the distribution and dynamic changes in the gut microbiota, aiming to further explore the regulatory effects of prophylactic BL-01 supplementation on UC. The Venn diagram showed that 2261 OTUs were shared among the three groups, whereas the CT, DSS, and BL-DSS groups had 242, 63, and 78 unique OTUs, respectively (Figure 4A). The Chao1 index and Simpson index were used to evaluate the α-diversity of the microbial community. Box plots showed that microbial diversity was decreased in the DSS group compared with the CT group, whereas it was increased in the BL-DSS group relative to the DSS group (Figure 4B,C). Although no statistically significant differences were observed, these findings highlight the potential of BL-01 to promote the restoration of microbial diversity. Similar results were observed in the β-diversity analysis. The principal coordinate analysis (PCoA) shows that the data points from each group were clustered separately; specifically, the DSS group was distinctly separated from the CT group, while the BL-DSS group was closely clustered with the CT group (Figure 4D). To further explore the community structure, we assessed the relative abundance of the dominant microbial taxa (top 20) classified at the phylum and genus levels. At the phylum level, the top three phyla in the CT and BL-DSS groups were *Bacteroidetes*, *Bacillota*, and *Verrucomicrobiota*, whereas those in the DSS group were *Bacteroidetes*, *Bacillota*, and *Campylobacterota* (Figure 4E). At the genus level, the DSS group showed increased abundances of *Helicobacter*, *Acetobacter*, and *Ruminococcus*, whereas the abundances of *Duncaniella*, *Prevotella*, *Muribaculum*, *Paramuribaculum*, and *Odoribacter* were decreased. In contrast, BL-01 supplementation reversed these DSS-induced alterations in the BL-DSS group (*p* < 0.05, *p* < 0.01, *p* < 0.001) (Figure 4F,G). The results of LEfSe and LDA were consistent with the above observations (LDA > 2) (Figure 4H). Collectively, these results indicate that BL-01 can restore DSS-induced gut microbiota dysbiosis by decreasing the abundance of harmful bacteria and increasing that of the beneficial bacteria.

### 3.5. Effects of BL-01 on Microbial Metabolic Processes of the Mouse Gut Microbiota

**Figure 5:**
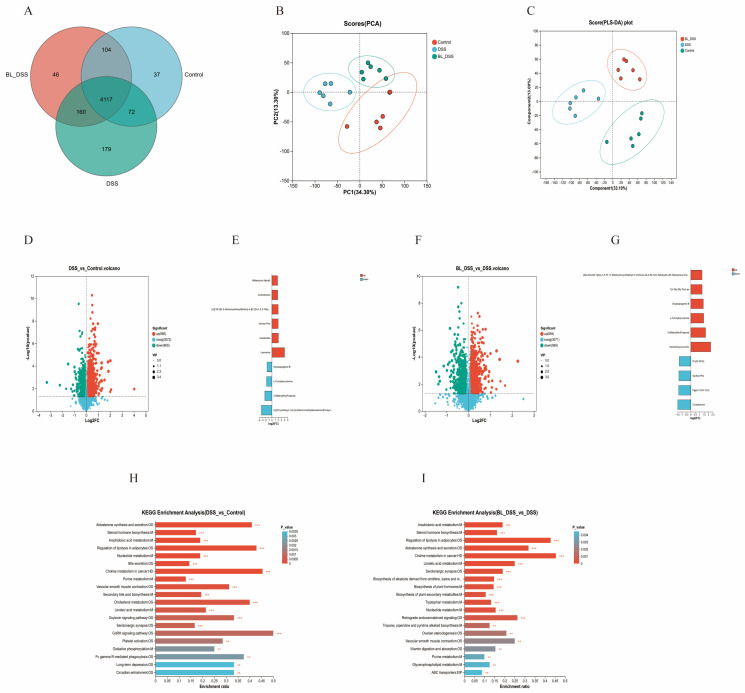
Metabolic profiling of fecal samples from DSS-induced colitis. (**A**) Venn diagram illustrating the overlap of metabolites among different groups. (**B**,**C**) PCA and PLS-DA score plots of gut metabolites showing the metabolite differences between groups. (**D**,**F**) Volcano plots highlighting significantly altered metabolites. (**E**,**G**) Top 10 metabolites ranked by the largest fold change. (**H**,**I**) KEGG enrichment analysis of differentially abundant metabolites. The data is provided in means + SD (*n* = 6). *** *p* < 0.001 and ** *p* < 0.01.

To elucidate the regulatory effects of prophylactic BL-01 supplementation on colonic microbial metabolism in mice with UC, a non-targeted metabolomics analysis of the colonic contents was performed. As shown in the Venn diagram, a total of 4117 metabolites were shared among the three groups. The CT group, DSS group, and BL-DSS group exhibited 37, 179, and 46 unique metabolites, respectively (Figure 5A). PCA was used to evaluate global metabolite changes. Notably, samples in the DSS group were clearly separated from those in the CT group, while samples in the BL-DSS group also showed distinct separation from those in the DSS group (Figure 5B). Meanwhile, PLS-DA revealed distinct metabolic profiles between the DSS group and the other two groups. In both ion modes, the DSS group was clearly separated from the CT and BL-DSS groups, indicating significant metabolite differences among these groups (Figure 5C). Subsequently, differentially expressed metabolites were identified using the criteria of *p* < 0.05 and VIP > 1. Compared with the CT group, the DSS group had 1462 differentially expressed metabolites, including 856 upregulated metabolites (including Leonurine, Avadomide, Val Asn Phe, 2-{[(1R,2S)-2-Aminocyclohexyl]Amino}-4-{[3-(2H-1,2,3-Triazol-2-Yl)Phenyl]Amino}Pyrimidine-5-Carboxamide, Cortodoxone, and Milbemycin Alpha9) and 606 downregulated metabolites (including 2-[[3-Cyclohexyl-1-[2-[3-(Diaminomethylideneamino)Propylcarbamoyl]Piperidin-1-Yl]-1-Oxopropan-2-Yl]Amino]Acetic Acid, 3-(Methylthio)Propanal, L-Formylkynurenine, and Soyasapogenol B) (Figure 5D,E). Compared with the DSS group, the BL-DSS group had 1463 differentially expressed metabolites, including 894 upregulated metabolites (including Demethoxycurcumin, 3-(Methylthio)Propanal, L-Formylkynurenine, Soyasapogenol B, and Tyr-Gly-Gly-Trp-Leu) and 569 downregulated metabolites (including Cortodoxone, Pgp(I-12:0/I-12:0), Val Asn Phe, and Pc(22:5/0:0)) (Figure 5F,G).

Subsequently, gene set enrichment analysis was performed using the KEGG database. To avoid false positive enrichment results and comply with the standard requirement for metabolomic enrichment analysis, the hypergeometric test was used as the statistical method for KEGG enrichment analysis, with the adjusted *p*-value (FDR) set at <0.05. Compared with the CT group, differentially expressed metabolites in the DSS group were primarily enriched in the following pathways: aldosterone synthesis and secretion, steroid hormone biosynthesis, arachidonic acid metabolism, regulation of lipolysis in adipocytes, and nucleotide metabolism (Figure 5H). Compared with the DSS group, differentially expressed metabolites in the BL-DSS group were mainly enriched in pathways including arachidonic acid metabolism, steroid hormone biosynthesis, the regulation of lipolysis in adipocytes, aldosterone synthesis and secretion, and choline metabolism in cancer (Figure 5I). The top four pathways were found to be co-regulated by DSS and BL-01. Among these four co-regulated pathways, a total of 18 differentially expressed metabolites were identified, including NAD+, DG(14:1(9Z)/16:1(9Z)/0:0), DG(15:0/16:0/0:0), Aldosterone, Adenosine Monophosphate, DG(14:0/20:3(8Z,11Z,14Z)/0:0), W-Hydroxy Testosterone, Dihydrocorticosterone, 17α,21-Dihydroxypregnenolone, 21-Hydroxy-5β-Pregnane-3,11,20-Trione, 16α-Hydroxydehydroisoandrosterone, Tetrahydrocorticosterone, 7α,17β-Dihydroxyandrost-4-en-3-one, 6-Keto-Prostaglandin F1A, Delta-12-Pgj2, Prostaglandin-C2, 11,12-Dihydrotetradecanedioic acid, and Tg(8:0/8:0/15:0).

## 4. Discussion

IBD is a worldwide disorder with rising cases, and it presents a significant risk to the health of the population [3]. Traditional treatments for IBD mainly rely on drugs but, unfortunately, the effectiveness of these drugs differ, and undesirable side effects are also a major problem. As a result, growing attention has been paid to developing safer and more effective therapeutic approaches, including dietary interventions and microbiome-based therapies [26]. Wang et al. established that blueberries have a therapeutic effect against DSS-induced UC through their endogenous anthocyanins by suppressing the endoplasmic reticulum stress-mediated apoptotic pathway [27]. Zheng et al. also demonstrated that *Bacteroides acidifaciens* can reduce colitis symptoms by preventing colonic mucosal damage, inflammatory responses, and regaining the diversity of gut microbiota. Moreover, *Bacteroides acidifaciens* extracellular vesicles are abundant in useful proteins that relieve colitis, thereby offering a new understanding of the pathways of microbial therapy of UC [11]. The present study shows that DSS-induced weight loss, diarrhea, and rectal bleeding were significantly attenuated by BL-01 intervention. Moreover, BL-01 is useful for stopping pathological changes such as colon shortening, a high DAI, and a high spleen index. These results support the potential use of BL-01 for the prevention of IBD.

Intestinal homeostasis and defense against external insults depend on the integrity of the mucosal barrier, and this barrier consists of mechanical, chemical, immunological, and biological elements [28]. In UC development, chemical barrier dysfunction and mechanical barrier dysfunction are the main markers of intestinal mucosal barrier dysfunction: goblet cell loss and reduced mucus secretion in the chemical barrier, and the downregulation of tight junction proteins in the mechanical barrier [29,30]. Critically, these barrier defects are largely driven by an imbalance of pro- and anti-inflammatory cytokines [31,32]. Pro-inflammatory cytokines (IL-1β, IL-6, TNF-α) directly cause intestinal inflammation and tissue damage, thereby accelerating UC progression and impeding mucosal healing, whereas anti-inflammatory cytokines (IL-4, IL-10) maintain barrier integrity by promoting epithelial proliferation and inhibiting apoptosis [33,34]. In the present DSS-induced colitis model, we observed goblet cell depletion and reduced the expression of tight junction proteins (ZO-1 and Occludin). These abnormalities were accompanied by an exaggerated release of pro-inflammatory cytokines and reduced expression of anti-inflammatory cytokines. Prophylactic BL-01 supplementation reversed all these changes, indicating that BL-01 restores normal bowel barrier function primarily by rebalancing the cytokine network, a well-recognized mechanism of probiotic immunomodulation. In addition, antioxidant imbalance and oxidative stress are well-established critical determinants of the pathogenesis of intestinal diseases [35]. Uncontrolled inflammatory responses lead to excessive production of ROS and free radicals, which perturb the oxidant–antioxidant equilibrium and ultimately compromise the integrity of the intestinal mucosal barrier [36,37]. This creates a vicious cycle: oxidative damage to intestinal epithelial cells amplifies inflammation, which in turn generates more ROS [38]. In the present study, the DSS group showed elevated serum MDA levels and decreased activities of antioxidant enzymes, including T-SOD, GSH-Px, and CAT. This indicates exacerbated lipid peroxidation and impaired antioxidant defense mechanisms in UC mice. In the BL-DSS group, these abnormalities were significantly alleviated, demonstrating that BL-01 can markedly enhance the host antioxidant defense capacity, mitigate oxidative damage, and thereby break the inflammation–oxidative stress cycle. It should be emphasized that the antioxidant parameters in the present study were determined in serum, rather than colonic tissue. Serum GSH-Px activity predominantly reflects the systemic extracellular antioxidant capacity, which is mainly attributed to secreted GPX3 in the circulation. Accordingly, it does not directly represent the colonic GPX activity, protein abundance, or gene expression [39,40,41]. Nevertheless, DSS-induced epithelial injury and mucosal inflammation are known to disrupt local colonic redox homeostasis, which in turn may lead to systemic alterations in circulating antioxidant enzyme activities in line with the severity of colonic damage [42]. Thus, the observed changes in serum GSH-Px activity are biologically plausible in the context of DSS colitis, even though they constitute an indirect, rather than a direct, measure of local tissue oxidative injury [43]. In addition, serum MDA levels should be interpreted cautiously, as they can be affected not only by systemic oxidative stress but also by microbiota-derived metabolic products and inflammatory mediators [44,45]. Moreover, conventional endpoint MDA measurements are subject to well-recognized analytical limitations [46]. Therefore, to enable a more robust mechanistic interpretation, future studies should directly evaluate oxidative injury in colonic tissue, combined with tissue-specific GPX activity and expression analyses.

The gut microbiota has recently garnered substantial scientific attention due to its critical role in maintaining human and animal health [47]. Accumulating evidence has revealed substantial alterations in gut microbial diversity and composition between healthy and diseased states, particularly in conditions such as IBD [48,49]. It has been demonstrated that gut microbiota dysbiosis contributes directly to the development of UC and worsens disease progression through a range of mechanisms that include metabolic impairment, destabilization of intestinal homeostasis, and interference with intestinal functionality [50]. Importantly, dysbiosis can be both a cause and a consequence of intestinal inflammation. In the present study, α- and β-diversity analyses were employed to evaluate microbial composition and diversity. Compared with the CT group, the DSS group exhibited significant alterations in microbial profiles. In contrast, the BL-DSS group showed greater similarity to the CT group, indicating that BL-01 has the ability to regulate gut microbiota dysbiosis. Furthermore, at the genus level, we observed that DSS treatment increased the abundance of *Helicobacter*, a genus strongly associated with appendicitis, colitis, and diarrhea [51]. Meanwhile, the abundance of *Ruminococcus*, a genus linked to inflammatory bowel disease, also increased significantly [52]. The abundance of both genera was found to be reduced with prophylactic BL-01 supplementation. At the same time, the DSS group demonstrated a significant reduction in the abundance of various beneficial bacterial genera, such as *Duncaniella*, *Prevotella*, and *Muribaculum*, and a significant increase in these genera was noted in the BL-DSS group [53,54,55]. Collectively, these results indicate that BL-01 can alleviate colonic inflammation by promoting the restoration of microbial homeostasis, which is achieved by replenishing beneficial bacterial species and reducing the abundance of potential pathogenic bacteria.

The gut microbiota is also capable of producing numerous metabolites that have particular biological purposes, which play a central role in the crosstalk between the gut bacteria and host cells [25,56]. These microbial-derived metabolites have been demonstrated to be closely linked to the composition and function of the gut microbiota. Alterations in the microbial community structure have been shown to directly alter the metabolic profiles of the host, while metabolic alterations can in turn feed back to regulate microbial growth and diversity. The metabolomic analysis showed that there are variations in the metabolic profiles of the groups, with significant differences in the BL-DSS and DSS groups showing that prophylactic BL-01 supplementation prevents the profound metabolic changes caused by DSS. In our results, we indicate that BL-01 intervention has the capacity to modulate pathways such as arachidonic acid metabolism and steroid hormone biosynthesis, which are closely associated with the microbial alterations. Arachidonic acid is a ω-6 polyunsaturated fatty acid that is found in cell membranes as phospholipids [57]. Arachidonic acid is also released under cellular stress conditions and converted into biologically active metabolites via the cyclooxygenase (COX), lipoxygenase (LOX), and cytochrome P450 (CYP450) pathways to enhance the inflammatory cascade [58]. According to Setty et al., the concentration of arachidonic acid has a negative relationship with the vaso-occlusive crises (VOC), one of the chronic inflammatory diseases in children with sickle cell anemia [59]. In the research by Madsen, the oxytocin metabolic pathway of pigs with gastric ulcers changed to pro-inflammatory arachidonic acid-derived eicosanoids, which may indicate a heightened inflammatory response [60]. In this study, we have found significant enrichment of the arachidonic acid metabolism pathway in the DSS group, with a significant increase in related metabolites findings that demonstrate the activation of the inflammatory response. It is important to note that this upregulation of arachidonic acid metabolism was closely associated with DSS-induced dysbiosis. The increased abundance of pro-inflammatory genera (*Helicobacter* and *Ruminococcus*) in the DSS group is likely to promote the release and metabolism of arachidonic acid, thereby exacerbating intestinal inflammation [61]. On the contrary, these metabolites were suppressed in the case of BL-01 intervention, and inflammation of the colon was alleviated. The same observation is in agreement with the findings of the detection of inflammatory markers in the serum. Steroid hormones control immune and inflammatory reactions through the adjustment of the metabolism of immune cells [62]. Cain et al. proposed that glucocorticoids can suppress the pro-inflammatory responses of Th1 and Th17 cells while enhancing the anti-inflammatory and immunosuppressive functions of Th2 and Treg cells [63]. Furthermore, Peng et al. observed a positive correlation between cortodoxone and T-cell immune responses [64]. During IBD progression, the continuous regeneration and repair of intestinal epithelial stem cells and intestinal mucosal epithelial cells are closely intertwined [65]. Meanwhile, immune cells, including T cells, have been shown to be intrinsically associated with the survival of intestinal epithelial stem cells [66,67]. This underscores the importance of the steroid hormone metabolism in inflammatory diseases. In our results, there was higher cortodoxone expression in the steroid hormone biosynthesis pathway in the DSS group, whereas the BL-01 intervention expression decreased. This metabolic change has also been linked to the microbial alterations induced by BL-01: the restored abundance of beneficial genera (*Duncaniella*, *Prevotella*, and *Muribaculum*) in the BL-DSS group may regulate steroid hormone biosynthesis by producing key metabolic intermediates or participating in the metabolism of steroid precursors, thereby restoring the balance of the steroid hormone metabolism and mitigating inflammation [68,69]. Taken together, these metabolic changes are not independent events, but rather downstream consequences of BL-01’s ability to rebalance the gut microbiota and dampen chronic inflammation. This supports the concept that BL-01 acts through a microbe–metabolite–immune axis, a recognized paradigm for probiotic action.

## 5. Conclusions

In conclusion, the current research presents thorough and methodical research involving the investigation of the protective influence of Bacillus licheniformis BL-01 in a mouse model of DSS-induced UC. The protective effects of BL-01 occur mainly via preserving the intestinal mucosal barrier, which is achieved through the inhibition of inflammatory cytokine secretion, the augmentation of host antioxidant capacity, and the suppression of oxidative damage. Moreover, it was also found that BL-01 reconfigures the gut microbiota, enhancing its diversity and richness and decreasing the presence of potentially pathogenic bacteria. Also, it was discovered that BL-01 stimulates the activation of major metabolic pathways and restores the expression of corresponding metabolites, thus ameliorating colonic inflammatory reactions. This article is one of a paucity of studies to demonstrate that Bacillus licheniformis alleviates intestinal inflammation and repairs the intestinal barrier by modulating the gut microbiota–metabolome axis. The study will contribute to our overall knowledge of the molecular pathways behind the therapeutic effect of BL-01 on DSS-induced UC, further expanding the selection of strains for precision microbiome therapy in IBD. These findings are highly relevant to human inflammatory bowel disease (IBD), as human ulcerative colitis shares similar pathological mechanisms, including intestinal barrier disruption, chronic inflammation, oxidative stress, and gut microbiota dysbiosis. This study provides experimental evidence supporting the translational potential of *Bacillus licheniformis* as a probiotic strategy for human IBD prevention and adjuvant treatment. Nevertheless, since BL-01 was used as a preventive medication, its efficacy as a treatment for diagnosed colitis requires further research to be verified.

## Data Availability

The original data presented in the study are openly available in NCBI at https://www.ncbi.nlm.nih.gov/bioproject/?term=PRJNA1429254 (accessed on 28 February 2026).

## References

[1] Tavakoli P., Vollmer-Conna U., Hadzi-Pavlovic D., Grimm M.C. (2021). A Review of Inflammatory Bowel Disease: A Model of Microbial, Immune and Neuropsychological Integration. Public Health Rev..

[2] Gajendran M., Loganathan P., Jimenez G., Catinella A.P., Ng N., Umapathy C., Ziade N., Hashash J.G. (2019). A comprehensive review and update on ulcerative colitis. Disease-a-Month.

[3] Kaplan G.G. (2015). The global burden of IBD: From 2015 to 2025. Nat. Rev. Gastroenterol. Hepatol..

[4] Nagayama M., Gogokhia L., Longman R.S. (2025). Precision microbiota therapy for IBD: Premise and promise. Gut Microbes.

[5] Stallmach A., Atreya R., Grunert P.C., Stallhofer J., de Laffolie J., Schmidt C. (2023). Treatment Strategies in Inflammatory Bowel Diseases. Dtsch. Arztebl. Int..

[6] Xie Q., Gong S., Cao J., Li A., Kulyar M.F., Wang B., Li J. (2024). Mesenchymal stem cells: A novel therapeutic approach for feline inflammatory bowel disease. Stem Cell Res. Ther..

[7] Filardo S., Di Pietro M., Sessa R. (2024). Current progresses and challenges for microbiome research in human health: A perspective. Front. Cell. Infect. Microbiol..

[8] Bethlehem L., Estevinho M.M., Grinspan A., Magro F., Faith J.J., Colombel J.F. (2024). Microbiota therapeutics for inflammatory bowel disease: The way forward. Lancet Gastroenterol. Hepatol..

[9] He Y., Li F., Zhang W., An M., Li A., Wang Y., Zhang Y., Fakhar E.A.K.M., Iqbal M., Li J. (2024). Probiotic Potential of *Bacillus amyloliquefaciens* Isolated from Tibetan Yaks. Probiotics Antimicrob. Proteins.

[10] Liu Q., Jian W., Wang L., Yang S., Niu Y., Xie S., Hayer K., Chen K., Zhang Y., Guo Y. (2023). Alleviation of DSS-induced colitis in mice by a new-isolated *Lactobacillus acidophilus* C4. Front. Microbiol..

[11] Zheng C., Zhong Y., Xie J., Wang Z., Zhang W., Pi Y., Zhang W., Liu L., Luo J., Xu W. (2023). *Bacteroides acidifaciens* and its derived extracellular vesicles improve DSS-induced colitis. Front. Microbiol..

[12] Xu M., Hu M., Han J., Wang L., He Y., Kulyar M.F., Zhang X., Lu Y., Mu S., Su H. (2024). The Therapeutic Effects of Lactic Acid Bacteria Isolated from Spotted Hyena on Dextran Sulfate Sodium-Induced Ulcerative Colitis in Mice. Nutrients.

[13] Zeng Z., Quan C., Zhou S., Gong S., Iqbal M., Kulyar M.F., Nawaz S., Li K., Li J. (2024). Gut microbiota and metabolic modulation by supplementation of polysaccharide-producing *Bacillus licheniformis* from Tibetan Yaks: A comprehensive multi-omics analysis. Int. J. Biol. Macromol..

[14] Shini S., Zhang D., Aland R.C., Li X., Dart P.J., Callaghan M.J., Speight R.E., Bryden W.L. (2020). Probiotic *Bacillus amyloliquefaciens* H57 ameliorates subclinical necrotic enteritis in broiler chicks by maintaining intestinal mucosal integrity and improving feed efficiency. Poult. Sci..

[15] Zeng Z., Zhang J., Li Y., Li K., Gong S., Li F., Wang P., Iqbal M., Kulyar M.F., Li J. (2022). Probiotic Potential of *Bacillus licheniformis* and *Bacillus pumilus* Isolated from Tibetan Yaks, China. Probiotics Antimicrob. Proteins.

[16] Li A., Wang Y., Li Z., Qamar H., Mehmood K., Zhang L., Liu J., Zhang H., Li J. (2019). Probiotics isolated from yaks improves the growth performance, antioxidant activity, and cytokines related to immunity and inflammation in mice. Microb. Cell Factories.

[17] Wang B., Wu Q., Yu S., Lu Q., Lv X., Zhang M., Kan Y., Wang X., Zhu Y., Wang G. (2023). Host-derived bacillus spp. as probiotic additives for improved growth performance in broilers. Poult. Sci..

[18] Sauer U., Treuner A., Buchholz M., Santangelo J.D., Dürre P. (1994). Sporulation and primary sigma factor homologous genes in *Clostridium acetobutylicum*. J. Bacteriol..

[19] Dong Y., Li R., Liu Y., Ma L., Zha J., Qiao X., Chai T., Wu B. (2020). Benefit of Dietary Supplementation with *Bacillus subtilis* BYS2 on Growth Performance, Immune Response, and Disease Resistance of Broilers. Probiotics Antimicrob. Proteins.

[20] Chen L., Li R., Wang Z., Zhang Z., Wang J., Qiao Y., Huang Y., Liu W. (2022). Lactate-utilizing bacteria ameliorates DSS-induced colitis in mice. Life Sci..

[21] Pan H., Yang S., Kulyar M.F., Ma H., Li K., Zhang L., Mo Q., Li J. (2025). *Lactobacillus fermentum* 016 Alleviates Mice Colitis by Modulating Oxidative Stress, Gut Microbiota, and Microbial Metabolism. Nutrients.

[22] Kim H.J., Jeon H.J., Kim J.Y., Shim J.J., Lee J.H. (2024). *Lactiplantibacillus plantarum* HY7718 Improves Intestinal Integrity in a DSS-Induced Ulcerative Colitis Mouse Model by Suppressing Inflammation through Modulation of the Gut Microbiota. Int. J. Mol. Sci..

[23] Wang Y., Xie Q., Zhang Y., Ma W., Ning K., Xiang J.Y., Cui J., Xiang H. (2020). Combination of probiotics with different functions alleviate DSS-induced colitis by regulating intestinal microbiota, IL-10, and barrier function. Appl. Microbiol. Biotechnol..

[24] Zhang Y., Ding Y., Mo Q., Kulyar M.F., He Y., Yao W., Quan C., Gong S., Li F., Fu Y. (2022). Sodium butyrate ameliorates thiram-induced tibial dyschondroplasia and gut microbial dysbiosis in broiler chickens. Ecotoxicol. Environ. Saf..

[25] Wang L., Zhang Z., Zhu X., Zhao Y., Iqbal M., Lin Z., Nawaz S., Xu M., Hu M., Bhutto Z.A. (2023). The Effect of *Lactobacillus sakei* on Growth Performance and Intestinal Health in Dogs: Gut Microbiota and Metabolism Study. Probiotics Antimicrob. Proteins.

[26] Liu H., Chen R., Wen S., Li Q., Lai X., Zhang Z., Sun L., Sun S., Cao F. (2023). Tea (*Camellia sinensis*) ameliorates DSS-induced colitis and liver injury by inhibiting TLR4/NF-κB/NLRP3 inflammasome in mice. Biomed. Pharmacother..

[27] Wang J., Wang X.Y., Yuan Z.Y., Wang X.H., Guan Y.Y., Zhu J.X., Huang W.F., Liu Q., Xu G.H., Yi L.T. (2024). Blueberry extract attenuates DSS-induced inflammatory bowel disease in mice through inhibiting ER stress-mediated colonic apoptosis in mice. Food Funct..

[28] Chen Y., Cui W., Li X., Yang H. (2021). Interaction Between Commensal Bacteria, Immune Response and the Intestinal Barrier in Inflammatory Bowel Disease. Front. Immunol..

[29] Kangwan N., Kongkarnka S., Boonkerd N., Unban K., Shetty K., Khanongnuch C. (2022). Protective Effect of Probiotics Isolated from Traditional Fermented Tea Leaves (Miang) from Northern Thailand and Role of Synbiotics in Ameliorating Experimental Ulcerative Colitis in Mice. Nutrients.

[30] Zhou X., Zhang Y., Hu M., Ge Z., Zhou G. (2023). Resveratrol enhances MUC2 synthesis via the ANRIL-miR-34a axis to mitigate IBD. Am. J. Transl. Res..

[31] Hu C., Liao S., Lv L., Li C., Mei Z. (2023). Intestinal Immune Imbalance is an Alarm in the Development of IBD. Mediat. Inflamm..

[32] Neurath M.F. (2024). Strategies for targeting cytokines in inflammatory bowel disease. Nat. Rev. Immunol..

[33] Borruel N., Carol M., Casellas F., Antolín M., de Lara F., Espín E., Naval J., Guarner F., Malagelada J.R. (2002). Increased mucosal tumour necrosis factor alpha production in Crohn’s disease can be downregulated ex vivo by probiotic bacteria. Gut.

[34] Ye M., Joosse M.E., Liu L., Sun Y., Dong Y., Cai C., Song Z., Zhang J., Brant S.R., Lazarev M. (2020). Deletion of IL-6 Exacerbates Colitis and Induces Systemic Inflammation in IL-10-Deficient Mice. J. Crohn’s Colitis.

[35] Muro P., Zhang L., Li S., Zhao Z., Jin T., Mao F., Mao Z. (2024). The emerging role of oxidative stress in inflammatory bowel disease. Front. Endocrinol..

[36] Huang Q.X., Liang J.L., Yang C.H., Li K., Niu M.T., Fan J.X., Zhang X.Z. (2023). Stimulation-responsive mucoadhesive probiotics for inflammatory bowel disease treatment by scavenging reactive oxygen species and regulating gut microbiota. Biomaterials.

[37] Zheng Y., Zhang L., Bonfili L., de Vivo L., Eleuteri A.M., Bellesi M. (2023). Probiotics Supplementation Attenuates Inflammation and Oxidative Stress Induced by Chronic Sleep Restriction. Nutrients.

[38] Vona R., Pallotta L., Cappelletti M., Severi C., Matarrese P. (2021). The Impact of Oxidative Stress in Human Pathology: Focus on Gastrointestinal Disorders. Antioxidants.

[39] Whitin J.C., Tham D.M., Bhamre S., Ornt D.B., Scandling J.D., Tune B.M., Salvatierra O., Avissar N., Cohen H.J. (1998). Plasma glutathione peroxidase and its relationship to renal proximal tubule function. Mol. Genet. Metab..

[40] Olson G.E., Whitin J.C., Hill K.E., Winfrey V.P., Motley A.K., Austin L.M., Deal J., Cohen H.J., Burk R.F. (2010). Extracellular glutathione peroxidase (Gpx3) binds specifically to basement membranes of mouse renal cortex tubule cells. Am. J. Physiol.-Ren. Physiol..

[41] Tham D.M., Whitin J.C., Kim K.K., Zhu S.X., Cohen H.J. (1998). Expression of extracellular glutathione peroxidase in human and mouse gastrointestinal tract. Am. J. Physiol..

[42] Tratenšek A., Locatelli I., Grabnar I., Drobne D., Vovk T. (2024). Oxidative stress-related biomarkers as promising indicators of inflammatory bowel disease activity: A systematic review and meta-analysis. Redox Biol..

[43] Tham D.M., Whitin J.C., Cohen H.J. (2002). Increased expression of extracellular glutathione peroxidase in mice with dextran sodium sulfate-induced experimental colitis. Pediatr. Res..

[44] Wu Z., Cheng W., Wang Z., Feng S., Zou H., Tan X., Yang Y., Wang Y., Zhang H., Dong M. (2021). Intestinal Microbiota and Serum Metabolic Profile Responded to Two Nutritional Different Diets in Mice. Front. Nutr..

[45] Unlü A., Türközkan N., Cimen B., Karabicak U., Yaman H. (2001). The effect of Escherichia coli-derived lipopolysaccharides on plasma levels of malondialdehyde and 3-nitrotyrosine. Clin. Chem. Lab. Med..

[46] Tsikas D. (2017). Assessment of lipid peroxidation by measuring malondialdehyde (MDA) and relatives in biological samples: Analytical and biological challenges. Anal. Biochem..

[47] Vascellari S., Palmas V., Melis M., Pisanu S., Cusano R., Uva P., Perra D., Madau V., Sarchioto M., Oppo V. (2020). Gut Microbiota and Metabolome Alterations Associated with Parkinson’s Disease. mSystems.

[48] Wang Y., Li A., Liu J., Mehmood K., Wangdui B., Shi H., Luo X., Zhang H., Li J. (2019). *L. pseudomesenteroides* and *L. johnsonii* isolated from yaks in Tibet modulate gut microbiota in mice to ameliorate enteroinvasive *Escherichia coli*-induced diarrhea. Microb. Pathog..

[49] Zhong S., Sun Y.-Q., Huo J.-X., Xu W.-Y., Yang Y.-N., Yang J.-B., Wu W.-J., Liu Y.-X., Wu C.-M., Li Y.-G. (2024). The gut microbiota-aromatic hydrocarbon receptor (AhR) axis mediates the anticolitic effect of polyphenol-rich extracts from Sanghuangporus. iMeta.

[50] Li M., Han X., Sun L., Liu X., Zhang W., Hao J. (2024). Indole-3-acetic acid alleviates DSS-induced colitis by promoting the production of R-equol from *Bifidobacterium pseudolongum*. Gut Microbes.

[51] Gravina A.G., Zagari R.M., De Musis C., Romano L., Loguercio C., Romano M. (2018). *Helicobacter pylori* and extragastric diseases: A review. World J. Gastroenterol..

[52] Martínez de Victoria Carazo J., Vinuesa García D., Serrano-Conde Sánchez E., Peregrina Rivas J.A., Ruíz Rodríguez A.J., Hernández Quero J. (2023). *Ruminococcus gnavus* bacteremia: Literature review and a case report associated with acute flare of ulcerative colitis in an immunocompromised patient. Anaerobe.

[53] Zhang J., Gao T., Chen G., Liang Y., Nie X., Gu W., Li L., Tong H., Huang W., Lu T. (2024). Vinegar-processed Schisandra Chinensis enhanced therapeutic effects on colitis-induced depression through tryptophan metabolism. Phytomed. Int. J. Phytother. Phytopharm..

[54] Yang C., Lan R., Zhao L., Pu J., Hu D., Yang J., Zhou H., Han L., Ye L., Jin D. (2024). *Prevotella copri* alleviates hyperglycemia and regulates gut microbiota and metabolic profiles in mice. mSystems.

[55] Xu H., Fang F., Wu K., Song J., Li Y., Lu X., Liu J., Zhou L., Yu W., Yu F. (2023). Gut microbiota-bile acid crosstalk regulates murine lipid metabolism via the intestinal FXR-FGF19 axis in diet-induced humanized dyslipidemia. Microbiome.

[56] Zhu D., Wang B., Xu Z., Yan Z., Kulyar M.F., Li S., Chen Y., Khateeb E., He S., Shen Y. (2025). Vitamin B(12)-producing *Cetobacterium*: An important biomarker linked to snake hibernation. Int. J. Biol. Macromol..

[57] Huss M., Elger T., Kunst C., Loibl J., Krautbauer S., Liebisch G., Kandulski A., Müller M., Tews H.C., Buechler C. (2025). Fecal Arachidonic Acid: A Potential Biomarker for Inflammatory Bowel Disease Severity. Int. J. Mol. Sci..

[58] Levick S.P., Loch D.C., Taylor S.M., Janicki J.S. (2007). Arachidonic acid metabolism as a potential mediator of cardiac fibrosis associated with inflammation. J. Immunol..

[59] Setty B.N.Y., Betal S.G., Miller R.E., Brown D.S., Meier M., Cahill M., Lerner N.B., Apollonsky N., Stuart M.J. (2019). Relationship of Omega-3 fatty acids DHA and EPA with the inflammatory biomarker hs-CRP in children with sickle cell anemia. Prostaglandins Leukot. Essent. Fat. Acids.

[60] Madsen P.A., Curtasu M.V., Canibe N., Hedemann M.S., Pedersen M.L.M., Lauridsen C. (2022). Non-targeted metabolomics of saliva to explore potential biomarkers for gastric ulceration in pigs fed hemp. Anim. Int. J. Anim. Biosci..

[61] Nakaya A., Wakabayashi H., Imamura L., Fukuta K., Makimoto S., Naganuma K., Orihara T., Minemura M., Shimizu Y., Nagasawa T. (2001). *Helicobacter pylori* alters n-6 fatty acid metabolism and prostaglandin E_2_ synthesis in rat gastric mucosal cells. J. Gastroenterol. Hepatol..

[62] Smith L.C., Ramar M., Riley G.L., Mathias C.B., Lee J.Y. (2025). Steroid hormone regulation of immunometabolism and inflammation. Front. Immunol..

[63] Zhu Y., Zhao Q., Huang Q., Li Y., Yu J., Zhang R., Liu J., Yan P., Xia J., Guo L. (2022). Nuciferine Regulates Immune Function and Gut Microbiota in DSS-Induced Ulcerative Colitis. Front. Vet. Sci..

[64] Peng C., Jiang X., Jaeger M., van Houten P., van Herwaarden A.E., Koeken V., Moorlag S., Mourits V.P., Lemmers H., Dijkstra H. (2024). 11-deoxycortisol positively correlates with T cell immune traits in physiological conditions. eBioMedicine.

[65] Neurath M.F., Artis D., Becker C. (2025). The intestinal barrier: A pivotal role in health, inflammation, and cancer. Lancet Gastroenterol. Hepatol..

[66] Hou Q., Huang J., Ayansola H., Masatoshi H., Zhang B. (2020). Intestinal Stem Cells and Immune Cell Relationships: Potential Therapeutic Targets for Inflammatory Bowel Diseases. Front. Immunol..

[67] Yokoi T., Murakami M., Kihara T., Seno S., Arase M., Wing J.B., Søndergaard J.N., Kuwahara R., Minagawa T., Oguro-Igashira E. (2023). Identification of a unique subset of tissue-resident memory CD4(+) T cells in Crohn’s disease. Proc. Natl. Acad. Sci. USA.

[68] Bai J., Gong Y., Ge J., Pei J., He Y., Zhang L., Min Y., Liu X., Yang X., Luo C. (2025). Egg matrices alleviate long-term florfenicol-induced behavioral alterations via the gut microbiota and host metabolism in mice. Food Funct..

[69] Fteita D., Könönen E., Gürsoy M., Ma X., Sintim H.O., Gürsoy U.K. (2018). Quorum sensing molecules regulate epithelial cytokine response and biofilm-related virulence of three *Prevotella* species. Anaerobe.

